# Atrophy of ventral diencephalon is associated with freezing of gait in Parkinson’s disease: analysis of two cohorts

**DOI:** 10.1038/s41531-025-00893-5

**Published:** 2025-03-06

**Authors:** Xuemei Wang, Huimin Chen, Xinxin Ma, Huijing Liu, Dongdong Wu, Wei Du, Jing He, Shuhua Li, Haibo Chen, Tao Wu, Tao Feng, Wen Su

**Affiliations:** 1 Center for Movement Disorders, Department of Neurology, Beijing Tiantan Hospital, Capital Medical University, Beijing, China; 2 Department of Neurology, Beijing Hospital, National Center of Gerontology, Institute of Geriatric Medicine, Chinese Academy of Medical Sciences, Beijing, China

**Keywords:** Parkinson's disease, Neurological manifestations, Brain

## Abstract

Evidence regarding brain structural atrophy associated with Freezing of Gait (FOG) in Parkinson’s disease (PD) is inconsistent. We analyzed cortical thickness and subcortical nuclei volumes using FreeSurfer in two large PD cohorts. In cohort 1 (*N* = 316), multivariate analyses identified reduced pallidum and ventral diencephalon (VDC) volumes as significantly associated with FOG presence. Validation in the Parkinson’s Progression Markers Initiative (PPMI) cohort (cohort 2, *N* = 94) demonstrated that decreased VDC volume at four-year follow-up independently predicted higher FOG risk, improving the predictive model’s accuracy when combined with PIGD score, CSF Aβ42, and caudate DAT uptake (AUC 0.760; Δ*χ*^2^ = 5.449, *P* = 0.020; *Z* = 2.211, *P* = 0.027). VDC volume is also correlated with FOG severity. These findings suggest that VDC atrophy may underlie FOG mechanisms and serve as a biomarker for its progression in PD patients.

## Introduction

Freezing of gait (FOG) is defined as an episodic absence or marked reduction in forward foot motion despite the intention to walk^1^. FOG affects 30–80% of patients with advanced Parkinson’s disease (PD)^2–4^, and significantly increases the risk of falls and reduces the quality of life in these patients. However, the precise mechanisms underlying FOG remain unclear.

Multiple studies have explored the brain structural changes underlying FOG development. Using volumetric analysis, Sunwoo and colleagues reported significant reduction in thalamic volume in PD-FOG^5^, whereas Canu et. al. found no significant gray matter change in PD-FOG^6^. A systematic review revealed considerable inconsistencies among studies concerning the exact region of brain atrophy affected by FOG; moreover, most were case-control studies with small sample sizes, often fewer than 50 patients^7^. Only one study by D’Cruz and colleagues investigated the longitudinal change in brain morphology in PD-FOG, and reported local inflations in bilateral thalamus in PD-FOG compared with PD-nFOG^8^, conflicting with Sunwoo’s study^5^. Additionally, a study by D’Cruz also had a small sample size, including only 12 patients with FOG at baseline and nine FOG converters during a 2-year follow-up. Further research with larger sample sizes and extended longitudinal studies were essential to determine if morphological changes are reliable biomarkers of PD-FOG and to understand better the neural mechanisms involved.

Parkinson’s Progression Markers Initiative (PPMI) is a large, ongoing, prospective cohort of PD. Earlier PPMI studies showed that baseline postural instability gait difficulty (PIGD) score, dopamine transporter (DAT) caudate uptake, and cerebrospinal fluid (CSF) Aβ42 levels predict FOG development over a four-year follow-up period^9,10^. Regarding brain structure, Li et al., reported that baseline cortical curvature in the occipital lobe, insula and supplementary motor cortex, and several white matter tracts predicted FOG development in the PPMI cohort^11^. The association between morphological alteration and FOG development during disease progression has been rarely investigated in the PPMI dataset. In the current study, we hypothesized that long-term morphological changes during disease progression may correlate with FOG development in PD.

This study included two cohorts: the first, a large, single-center consecutive cohort of PD patients undergoing magnetic resonance imaging (MRI) with three-dimensional T1 sequence (3D-T1) (cohort 1, *N* = 316); the second, a longitudinal cohort of FOG-free PD patients with eligible 3D-T1 at baseline and 4-year follow-up from PPMI (cohort 2, *N* = 94). We first conducted a cross-sectional analysis of PD-FOG-related morphological abnormalities using a data-driven approach in cohort 1; then, we tested whether these morphological abnormalities were associated with FOG development during the four-year follow-up in cohort 2. This study will provide more evidence concerning FOG-related brain structural change in patients with PD.

## Methods

### Patient selection

Cohort 1 comprised a single-center, consecutive, prospective group of 316 patients with idiopathic PD who underwent MRI scans with a 3D-T1 sequence at the Center for Movement Disorders, Department of Neurology, Beijing Tiantan Hospital from October 2018 to January 2020. Patients were diagnosed with clinically established or clinically probable PD based on the Movement Disorder Society clinical diagnostic criteria^12^. A reported good response to levodopa or >30% improvement in response to levodopa was required to differentiate PD from atypical parkinsonism. Exclusion criteria included: (1) uncertain PD diagnosis or suspicion of other parkinsonism syndrome (e.g., vascular, drug-induced, toxin-induced, postinfectious parkinsonism), multiple system atrophy, corticobasal ganglionic degeneration, or progressive supranuclear palsy; (2) a history of moderate-to-severe head trauma, hydrocephalus, brain surgery, or brain tumor; and (3) an inability to cooperate or communicate. This study was approved by the Ethics Committee of the Beijing Tiantan Hospital, and was performed in accordance with the Declaration of Helsinki. Informed consent was obtained either from the participants or their closest relatives.

Cohort 2: PPMI is an ongoing, multicenter, longitudinal, observational study that was initiated in 2010 (https://www.ppmi-info.org/). Patients with drug naïve PD were included. The specific inclusion and exclusion criteria are detailed elsewhere^13^. The PPMI study was approved by the institutional review board at each site, and participants provided written informed consent to participate. From the entire PPMI cohort, 94 FOG-free patients who had complete 3D-T1 images with 1 × 1 × 1 mm³ spatial resolution at both baseline and year four were selected for the current analysis (see flowchart in Supplementary Fig. 1). The data utilized in this study were downloaded from the PPMI dataset in June 2023.

### Clinical assessment

Cohort 1: Demographic profiles, including age, sex, and education duration, were recorded at admission. The motor severity of PD was assessed using the Hoehn and Yahr stage and the Movement Disorder Society Unified Parkinson’s Disease Rating Scale part III (MDS-UPDRS III). Cognition was evaluated using the Mini-Mental State Examination (MMSE) and Montreal Cognitive Assessment (MoCA). Psychological symptoms were assessed using a 14-item Hamilton Anxiety Rating Scale (HAMA)^14^ and a 24-item Hamilton Depression Rating Scale (HAMD)^15^. Levodopa daily dosage was recorded as the sum of daily levodopa intake derived from dopaminergic medications such as Madopar and Sinemet. FOG was evaluated using the Freezing of Gait Questionnaire (FOGQ)^16^, with the presence of FOG defined by a score ≥1 on item 3 of the FOGQ. All the patients had their motor status examined during off-state, while the tests of MMSE, MoCA, HAMA, and HAMD were not specified for on- or off-state. The severity of FOG was assessed by a total score of FOGQ item 3 (range 0–4).

Cohort 2: For the PPMI cohort, FOG was assessed by MDS-UPDRS item 2.13 (freezing) and item 3.11 (FOG). A FOG converter was defined as patients who scored 0 on both items at baseline but scored ≥1 on either item at any visit (every six months) during the 4-year follow-up period^9^. The severity of FOG was assessed by the sum score of MDS-UPDRS item 2.13 and item 3.11 (range 0–8). Motor function was assessed using the MDS-UPDRS III and Hoehn–Yahr stage. Overall cognition at baseline was evaluated by MoCA score. PIGD score was calculated by the summation of MDS-UPDRS item 2.12 (walking and balance), 3.10 (gait), and 3.12 (postural stability)^10^. DAT single-photon emission computed tomography (SPECT) imaging was acquired at PPMI imaging centers in accordance with the PPMI imaging protocol. Mean caudate and putaminal uptakes relative to uptake in the occipital area (striatal binding ratio [SBR]) using DAT-SPECT were computed. For CSF biomarkers, the concentration of α-syn in CSF samples was analyzed using an ELISA assay available commercially from BioLegend. CSF Aβ42, total tau (tTau), and tau phosphorylated at the threonine 181 position (pTau) were analyzed at Biorepository Core laboratories at the University of Pennsylvania using Elecsys electrochemiluminescence immunoassays (Roche Diagnostics). Further details on the protocol, CSF biomarkers, clinical variables, and imaging paradigm have been described in previous publications^13^.

### MRI data acquisition and processing

Cohort 1: Neuroimage was acquired on 3-Tesla MR scanners (Philips Ingenia CX, the Netherlands). Sagittal 3D-T1 images were acquired using the Magnetization Prepared-RApidGradient Echo imaging (MPRAGE) sequence (TR = 6.6 ms, TE = 3 ms, TI = 880 ms, flip angle = 8°, and image resolution = 1 × 1 × 1 mm^3^, acquisition matrix = 240 × 240).

Cohort 2: MRI images of the included subjects were acquired on Tim Trio 3-Tesla Siemens scanners (Siemens, Erlangen, Germany). Sagittal 3D-T1 weighted images were acquired using accelerated MPRAGE sequence: TR = 2300 ms, TE = 2.98 ms, flip angle = 9°, image resolution = 1.0 × 1.0 × 1.0 mm^3^, acquisition matrix = 240 × 256.

For both cohorts, all images were analyzed using the automated and validated pipeline “recon-all” implemented in FreeSurfer (version 6.0.0)^17^. Briefly, the processing pipeline consists of 34 stages described in the help document of “recon-all”, the main stages of which include normalization of brain signal intensity, skull-stripping, white matter and gray matter segmentation, and delineation of the gray-white interface (inner surface) and the pial surface (outer surface). Next, the surface is divided into separate cortical regions using an automated labeling approach, where not only location information based on the probabilistic surface-based atlas, but also local curvature and contextual information (e.g., sulcal and gyral geometry) of subject-specific surface are taken into consideration. Finally, surface area and mean cortical thickness were extracted for each of the 68 cortical regions (34 per hemisphere)^18^, and volume of 16 subcortical nucleus (the bilateral thalamus, putamen, caudate, pallidum, ventral diencephalon [VDC], amygdala, accumbens area, hippocampus) and five subregions of cingulate cortex (the anterior, mid-anterior, central, mid-posterior, and posterior cingulate cortex) were automatically segmented in FreeSurfer pipeline and extracted in the parcellation scheme^17^. Calculations were made in each subject’s native space.

### Statistical analysis

Categorical variables were presented as percentages, and continuous variables as means with standard deviation (SD) or medians with interquartile ranges, depending on their distribution. Group comparisons utilized the *χ*^2^ test for categorical variables and the two-sample *t*-test or Mann–Whitney *U*-test for continuous variables.

In cohort 1, to assess the FOG-related pattern of regional atrophy, principal component analysis (PCA) was initially conducted in the whole PD population to reduce the dimensionality of the 89 regional cortical thicknesses and subcortical volumes. The Bartlett test of sphericity and the Kaiser–Meyer–Olkin (KMO) measure were calculated to verify the appropriateness of PCA. Principal components (PCs) accounting for more than 5% of the variance in brain structure parameters were selected for further analysis. Regions with a correlation coefficient *R* > 0.7 within the component matrix were identified as major contributors to each PC. Subsequently, multivariate logistic regression was applied with FOG presence as the dependent variable, PC scores as independent variables, and demographic factors showing a trend toward significant difference (*P* < 0.1) as covariates. Specific morphological parameters contributing to FOG-associated PCs were compared between PD-FOG and PD-nFOG groups, and their relationships with PD-FOG were examined using logistic regression models. Given the significant intercorrelations among subcortical nucleus volumes, they were entered into models separately to prevent collinearity. Pearson correlation of selected PC scores with covariates was conducted to test collinearity. The odds ratio (OR), 95% confidence intervals (CIs), and uncorrected *P* values (*P*-raw) were documented. The false discovery rate (FDR) correction, via the Benjamini–Hochberg method, was employed for multiple comparisons, with a corrected *P* value (*P* cor) <0.05 deemed significant for brain structure variables.

In cohort 2 (PPMI cohort), baseline predictors of FOG development identified in prior PPMI study (i.e., PIGD score, mean caudate DAT uptake, CSF Aβ42 level)^9^ were also chosen as variables and tested in the present analysis. The subcortical volumes identified in Cohort 1 were examined in Cox regression models in the PPMI cohort. The hazard ratio (HR), 95% CI, and *P* value were reported for each variable. Model performance was assessed by the area under the curve (AUC) value, the absolute *χ*2 value, the change in *χ*^2^, and the *P* value for the change in *χ*^2^, and models were compared using the Delong test. Significance was established at a *P* value <0.05 (two-sided). Univariate and multivariate linear regression models, Spearman correlation, and partial correlation analysis were conducted for the association between FOG-related morphological parameters and FOG severity in both cohorts. For multivariate linear regression and partial correlation analysis, variables showing a trend toward significant association in univariate linear regression analysis were used as covariates.

Analyses were performed using SPSS 25.0 (SPSS, Inc., Chicago, IL, USA) and R software 4.3.1 (http://cran.r-project.org/).

## Results

### Group comparison between PD-FOG and PD-nFOG in cohort 1

The study consecutively included 316 patients with PD, and 153 (48.4%) patients were classified as PD-FOG. All included patients underwent an MRI scan with a 3D-T1 image. Compared with PD-nFOG, patients with FOG were older, had longer disease duration, higher Hoehn–Yahr stage, more impaired motor function, and higher levodopa daily dosage (Table 1).

**Table 1:** Demographic comparison between PD-FOG and PD-nFOG groups in cohort 1

Characteristics	PD-FOG (*N* = 153)	PD-nFOG (*N* = 163)	*P* value
Age at admission, years, median (IQR)	69 (60–75)	65 (57–69)	**0.002**
Sex, *n* (F/M)	66/87	68/95	0.799
BMI, kg/m^2^, mean ± SD	24.15 ± 3.07	24.05 ± 3.09	0.772
Disease profiles			
Disease duration, months, median (IQR)	7 (4–10)	5 (3–9)	**0.001**
Hoehn–Yahr stage, median (IQR)	3 (2.5–4)	3 (2–3)	**<0.001**
Levodopa daily dosage at admission, mg, median (IQR)	500 (300–687.5)	300 (200–545.5)	**<0.001**
MDS-UPDRS III, median (IQR)	46 (28–60)	36 (25–48)	**<0.001**
MMSE, median (IQR)	27 (22–29)	27 (25–29)	0.564
MoCA, median (IQR)	21 (17–25)	22 (19–25)	0.066
HAMA, median (IQR)	9 (5–14)	8 (5–12)	0.131
HAMD, median (IQR)	9 (6–13)	8 (5–14)	0.341
White matter hyperintensity			
Periventricular Fazekas rating scale, median (IQR)	1 (0–2)	1 (0–2)	0.780
Deep Fazekas rating scale, median (IQR)	1 (1–2)	1 (1–1)	0.120
FOG severity, median (IQR)	3 (2–4)	0 (0–0)	**<0.001**

*BMI* body mass index, *IQR* interquartile range, *HAMA* Hamilton anxiety scale, *HAMD* Hamilton depression scale, *MDS-UPDRS III* movement disorder society united Parkinson’s disease rating scale part III, *MMSE* mini-mental state examination, *MoCA* Montreal cognitive assessment, *SD* standard deviation.

Bold values identify statistical significance (*P* < 0.05).

### Principal component regression in cohort 1

The thickness of 68 cortical regions and volumes of 16 subcortical nucleus and five subregions of cingulate cortex were calculated. Out of these 89 structural parameters, 17 components were generated by PCA (Supplementary Fig. 2). *P* value of Bartlett test of sphericity <0.001 and Kaiser–Meyer–Olkin (KMO) = 0.917, suggesting that PCA was applicable. PC1 to PC17 accounted for 30.94, 10.71, 5.53, 3.27, 2.90, 2.54, 2.10, 1.90, 1.64, 1.60, 1.55%, 1.43, 1.35, 1.30, 1.21, 1.18, and 1.14% variance of brain structure. Only PCs accounting for >5% variance were chosen as independent factors for further regression analysis.

Logistic regression analysis using FOG presence as the outcome parameter showed that lower PC2 score was significantly associated with a higher prevalence of FOG in both the unadjusted model and the model adjusting for age, disease duration, Hoehn–Yahr stage, MDS-UPDRS III, MoCA, and levodopa dosage (Table 2). PC2 was not correlated with age (*R* = −0.063, *P* = 0.261), disease duration (*R* = 0.018, *P* = 0.751), Hoehn–Yahr stage (*R* = −0.074, *P* = 0.191), MDS-UPDRS III (*R* = 0.035, *P* = 0.542), MoCA (*R* = 0.008, *P* = 0.893) or levodopa dosage (*R* = −0.057, *P* = 0.312), suggesting absence of collinearity of PC2 with covariates.

**Table 2:** PC regression in cohort 1

	Model 1		Model 2	
	OR (95% CI)	*P* value	OR (95% CI)	*P* value
PC1	0.916 (0.733–1.145)	0.442	0.902 (0.699–1.164)	0.428
PC2	0.779 (0.621–0.977)	**0.031**	0.735 (0.568–0.951)	**0.019**
PC3	0.979 (0.784–1.224)	0.855	1.025 (0.796–1.320)	0.849
Age	1.029 (1.006–1.052)	**0.014**	1.018 (0.991–1.046)	0.187
Disease duration	1.006 (1.002–1.010)	**0.006**	0.998 (0.993–1.003)	0.502
Hoehn–Yahr stage	2.305 (1.702–3.121)	**<0.001**	1.490 (1.009–2.200)	**0.045**
MDS-UPDRS III	1.034 (1.020–1.048)	**<0.001**	1.023 (1.006–1.040)	**0.009**
Levodopa daily dosage	1.002 (1.001–1.003)	**<0.001**	1.002 (1.001–1.003)	**0.003**
MoCA	0.971 (0.935–1.009)	0.128	1.017 (0.975–1.061)	0.428

Model 1 was unadjusted.

Model 2 adjusted for age, disease duration, Hoehn–Yahr stage, MDS-UPDRS III, MoCA, and levodopa dosage. All parameters entered the models simultaneously.

*CI* confidence interval, *OR* odds ratio, *PC* principal component, *MDS-UPDRS III* movement disorder society united Parkinson’s disease rating scale part III, *MoCA* Montreal cognitive assessment.

Bold values identify statistical significance (*P* < 0.05).

PC2 was mainly composed of volumes of subcortical nucleus, including the left thalamus (*R* = 0.701), the left putamen (*R* = 0.735), the left pallidum (*R* = 0.752), the left VDC (*R* = 0.765), the right thalamus (*R* = 0.729), the right putamen (*R* = 0.734), the right pallidum (*R* = 0.781), and the right VDC (*R* = 0.757) (Supplementary Fig. 2).

### Association between subcortical volumes and PD-FOG in cohort 1

**Fig. 1:**
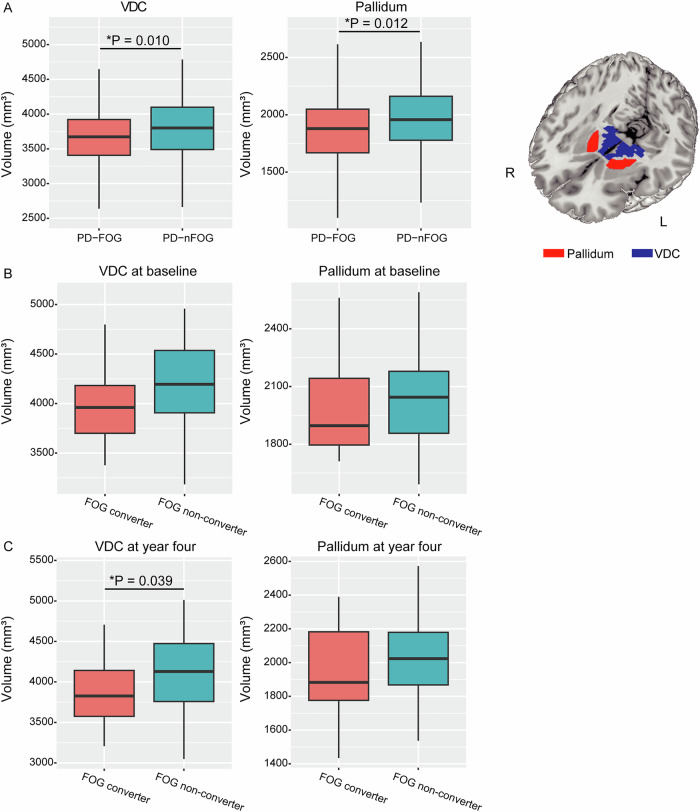
Comparison of subcortical volumes between FOG groups in two cohorts. **A** Comparison of VDC and pallidum volumes between patients with and without FOG in cohort 1. **B** Comparison of VDC and pallidum volumes at baseline between patients with and without FOG development in PPMI cohort. **C** Comparison of VDC and pallidum volumes at four-year follow-up between patients with and without FOG development in PPMI cohort. FOG freezing of gait, VDC ventral diencephalon.

The bilateral mean volumes of the eight subcortical nucleus (the bilateral thalamus, putamen, pallidum, VDC), which comprised PC2, were compared between PD-FOG and PD-nFOG groups. Result showed that volumes of pallidum (PD-FOG 1877.07 ± 823.23 VS. PD-nFOG 1963.95 ± 836.64 mm^3^, *P* = 0.012) and VDC (PD-FOG 3681.02 ± 424.13 VS. PD-nFOG 3804.61 ± 419.64 mm^3^, *P* = 0.010) were significantly lower in PD patients with FOG compared with PD-nFOG (Fig. 1A).

The difference in volume of the thalamus (PD-FOG 6324.20 ± 823.23 VS. PD-nFOG 6498.21 ± 836.64 mm^3^, *P* = 0.064) between groups was marginally significant. The volume of putamen (PD-FOG 4450.34 ± 761.18 VS. PD-nFOG 4536.83 ± 662.70 mm^3^, *P* = 0.281) was not significantly different between groups statistically.

In the multivariate logistic regression model, only the volumes of pallidum and VDC were still significantly associated with PD-FOG with adjustments (Table 3).

**Table 3:** The association between specific subcortical volumes and FOG in cohort 1

	Model 1			Model 2		
	OR (95% CI)	*P* raw value	*P* cor value	OR (95% CI)	*P* raw value	*P* cor value
Thalamus	1.000 (0.999–1.000)	0.106	0.141	1.000 (0.999–1.000)	0.147	0.159
Pallidum	0.999 (0.998–1.000)	**0.004**	**0.016**	0.999 (0.998–1.000)	**0.004**	**0.016**
VDC	0.999 (0.999–1.000)	**0.011**	**0.022**	0.999 (0.999–1.000)	**0.016**	**0.032**
Putamen	1.000 (0.999–1.000)	0.156	0.156	1.000 (0.999–1.000)	0.159	0.159

Model 1 adjusted for Hoehn–Yahr staging, levodopa daily dosage, MDS-UPDRS III.

Model 2 adjusted for age, disease duration, Hoehn–Yahr staging, levodopa daily dosage, MDS-UPDRS III.

*CI* confidence interval, *VDC* ventral diencephalon, *MDS-UPDRS III* movement disorder society united Parkinson’s disease rating scale part III, *OR* odds ratio.

Bold values identify statistical significance (*P* < 0.05).

### Validation analysis in the PPMI cohort

A total of eligible 94 patients with qualified 3D-T1 images at both baseline and four-year follow-up in the PPMI cohort were included in the present analysis (Supplementary Fig. 1). The comparison of the included patients (*N* = 94) and the excluded patients (*N* = 329) showed similar demographic and disease profiles (Supplementary Table 1). Compared with FOG non-converters, FOG converters showed significantly lower VDC volume (converter 3923.00 ± 480.51 VS. nonconverter 4136.68 ± 445.64 mm^3^, *P* = 0.039) at four-year follow-up. The baseline volume of VDC showed a marginal difference between FOG converters and non-converters (4018.85 ± 420.48 VS. 4185.24 ± 424.19 mm^3^, *P* = 0.081). The volume of pallidum either at baseline (converter 1988.63 ± 225.30 VS. nonconverter 2047.65 ± 219.54 mm^3^, *P* = 0.235) or at four-year follow-up (converter 1961.15 ± 252.99 VS. nonconverter 2027.23 ± 228.32 mm^3^, *P* = 0.213) did not show significant group difference (Fig. 1B, C).

**Fig. 2:**
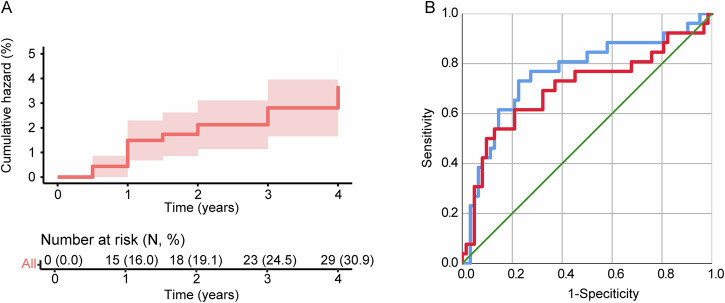
Kaplan–Meier curve and ROC curve of predictive models. **A** Kaplan–Meier curve for a cumulative incidence of FOG during 4-year follow-up in the included PPMI dataset; **B** Adding the volume of VDC at year four (blue line) improved the performance of model 1 (red line) for the prediction of FOG conversion (AUC = 0.760 [0.641–0.879] VS. 0.701 [0.567–0.835]). AUC area under curve, FOG freezing of gait, PIGD postural instability gait difficulty, ROC receiver operating characteristic, VDC ventral diencephalon.

Among the included subjects, the cumulative incidence of FOG was 16.0, 19.1, 24.5, and 30.9% annually (Fig. 2A). The conversion rate was similar to previous publication^9^. The PIGD score, mean caudate uptake, and CSF Aβ42 level were predictive of FOG development in the current subset of PPMI data (FOG risk = 0.514 × PIGD score-0.002 × CSF Aβ-1.315 × mean caudate DAT uptake), in consistence with previous publication (Table 4). In addition, the result showed that VDC volume at four-year follow-up was also significantly associated with FOG development (*P* < 0.05), after adjusting for PIGD score, mean caudate uptake, and CSF Aβ42 level. Adding the VDC volume at four-year follow-up to the model (FOG risk = 0.567 × PIGD score-0.002 × CSF Aβ-1.520 × mean caudate DAT uptake-0.001 × VDC volume at year four) improved model fit with statistical significance (Change in *χ*^2^ = 5.449, *P*_for change_ = 0.020, AUC = 0.760 [0.641–0.879] VS. 0.701 [0.567–0.835]; Delong test *Z* = 2.211, *P* = 0.027) (Table 4 and Fig. 2B). VDC volume at year four was not significantly correlated with PIGD score (*R* = −0.071, *P* = 0.495), CSF Aβ level (*R* = 0.018, *P* = 0.863), or mean caudate DAT uptake (*R* = −0.086, *P* = 0.417), suggesting the absence of collinearity.

**Table 4:** Cox regression models in the PPMI cohort

	HR (95% CI)	*P* value	χ2 (df, *P* value)	Change in χ2 (*P*_for change_)	Delong test
Model 1			34.198 (df = 3, ***P*** < **0.001**)	NA	NA
PIGD score	1.673 (1.296–2.160)	**<0.001**			
Mean caudate uptake	0.268 (0.121–0.595)	**0.001**			
CSF Aβ42	0.998 (0.997–1.000)	**0.019**			
Model 2 = Model 1+ volume of VDC at four-year follow-up			37.171 (df = 4, ***P*** < **0.001**)	5.449 (*P*_**for change**_ = **0.020**)	Z = 2.211, ***P*** = **0.027**
PIGD score	1.764 (1.349–2.306)	**<0.001**			
Mean caudate uptake	0.219 (0.097–0.492)	**<0.001**			
CSF Aβ42	0.998 (0.996–0.999)	**0.006**			
VDC volume at four-year follow-up	0.999 (0.998–1.000)	**0.024**			

*CI* confidence interval, *CSF* cerebrospinal fluid, *HR* hazard ratio, *PIGD* postural instability gait difficulty.

Bold values identify statistical significance (*P* < 0.05).

### Association between VDC volume and FOG severity

In cohort 1, the Spearman correlation did not show a significant correlation between FOG severity and VDC volume (*R* = −0.106, *P* = 0.119). VDC volume was significantly associated with FOG severity in univariate linear regression (unstandardized β = 0 [−0.001–0], *P* = 0.039), but not in multivariate linear regression (Supplementary Table 2) and partial correlation analysis (*R* = -0.097, *P* = 0.159) after adjusting for age, disease duration, MDS-UPDRS III, levodopa daily dosage, Hoehn–Yahr stage.

In PPMI cohort, the Spearman correlation showed a significant correlation between FOG severity and VDC volume at year four (*R* = −0.280, *P* = 0.006). Univariate and multivariate linear regression analyses demonstrated that VDC volume at year four was significantly associated with FOG severity (Supplementary Table 3). Partial correlation analysis yielded a similar result (*R* = −0.230, *P* = 0.032) after adjusting for age, disease duration, MDS-UPDRS III, and mean caudate uptake.

## Discussion

This study initially investigated FOG-related brain atrophy in a cohort of 316 PD patients (cohort 1) and subsequently validated these findings using longitudinal data from the PPMI cohort (cohort 2). We found that the volume of VDC was cross-sectionally associated with the presence of FOG in cohort 1. Furthermore, VDC volume at the four-year follow-up showed a significant association with FOG development in the PPMI cohort. In addition, VDC volume at four-year follow-up was associated with FOG severity in the PPMI cohort. The findings suggested that VDC atrophy may underlie the mechanism of FOG development and could serve as a biomarker for FOG in PD patients.

Firstly, our findings demonstrated an association between VDC volume and the presence or development of FOG in both cohorts studied. VDC encompasses several critical brain structures relevant to PD, including the subthalamic nucleus (STN), substantia nigra, basal forebrain, zona incerta, and adjacent white matter tracts. It has been well established that synucleinopathy in the substantia nigra, a pathological hallmark of PD, leads to dopaminergic deficiency in the striatum. This deficiency may enhance inhibitory outputs from the STN and the internal segment of the globus pallidus (GPi) to the thalamo-cortical and pedunculopontine nucleus circuits, potentially contributing to the FOG phenomenon^19,20^. While this model is supported by various studies, the specific contributions of the STN and its neighboring regions (such as zona incerta and Forel’s fields) remain incompletely defined. For instance, emerging evidence suggested that deep brain stimulation at zona incerta^21^, or the targeting of the thalamic fasciculus within the VDC, may provide therapeutic benefits comparable to those achieved by stimulating the STN directly^22–24^. Moreover, the basal forebrain, located within VDC, is a crucial component of the brain’s cholinergic system. Atrophy of the basal forebrain has been associated with cognitive decline and gait disturbances, including FOG, in PD^25–27^. The FOG-related VDC volume loss may reflect the atrophy of these nucleus within VDC and the loss of their projecting fibers. Whether cumulative changes in these structures within the VDC offer a more sensitive biomarker than alterations in a single structure remains to be tested in future studies.

In our study, VDC volume at the four-year follow-up, rather than baseline measurements, was significantly associated with FOG development and FOG severity in the PPMI cohort. Furthermore, the patients assessed in the cross-sectional analysis in cohort 1 predominantly had advanced PD, as indicated by a median Hoehn–Yahr stage of 3. This suggests that VDC atrophy may represent a later manifestation in the disease’s progression, rather than serving as an early biomarker. Indeed, prior research has frequently identified early changes in functional activity and connectivity as precursors to FOG development in PD, whereas morphological changes related to FOG at the early disease stage are less documented^28^. This is consistent with the pathophysiological process of PD, where brain atrophy is uncommon at early disease stage despite of significant loss of dopaminergic neurons and profound motor dysfunction^29^.

It should be noted that although the association between the volume of pallidum and the development of FOG did not reach significance in the PPMI cohort, it was statistically significant in the cross-sectional analysis in cohort 1. Our previous study using diffusion tensor imaging found altered microstructure in pallidum in PD-FOG compared with PD-nFOG, which was also correlated with FOG severity^30^. The role and long-term morphological change of pallidum warrants further investigation with a larger sample size.

There are some strengths of the study. First, two independent cohorts were included with large sample sizes and longitudinal observation, enabling reliable and cross-validated results. Second, a data-driven approach (i.e., PCA) was used to assess the association between regional brain atrophy and PD-FOG to minimize subjective selection or potential neglect of certain brain regions. We also acknowledge several limitations. First, the included PPMI subjects were a subset of the previous publication^9^ due to missed follow-up MRI images, which could potentially reduce the statistical power and representativeness of the present dataset. However, comparison between included and excluded subjects showed balanced demographic and disease profiles at baseline; in addition, the predictive model for FOG development using the current PPMI dataset was consistent with previous publications using the whole PPMI cohort^9^. These results suggested that the included 94 patients of PD were representative of the whole PPMI population. Nevertheless, some conclusions (e.g., the association between pallidum atrophy and FOG development) still need to be drawn with caution due to reduced statistical power. Second, the VDC is an obscure structure with multiple nucleus and white matter tracts, and the exact structure within VDC that is involved was not investigated in the present study. Based on the pathophysiology of FOG, the morphology and microstructure of STN, substantia nigra, zona incerta, basal forebrain, and fibers connecting with them warrant future investigations with high-resolution and multimodal neuroimaging techniques. Third, VDC atrophy at the advanced disease stage makes it an unsuitable biomarker for prodromal research. More studies are needed to find early functional or microstructural abnormality predictive of the onset of FOG in PD. Fourth, VDC volume was significantly associated with FOG severity only in the PPMI cohort but not in cohort 1, which may be due to the narrow range of FOGQ item 3 and its low discriminability for FOG severity. The lack of full FOGQ for the evaluation of FOG severity was another limitation of our study.

VDC atrophy may serve as a potential mechanism and a biomarker for FOG in PD.

## Supplementary information

Supplementary Material

## Data Availability

All data generated or analyzed during this study are included in this article. Further enquiries can be directed to the corresponding author. Data of cohort 1 was available upon reasonable request. PPMI data were publicly available on the PPMI website (https://www.ppmi-info.org/access-data-specimens/).
