# Supplementary material for: Atrophy of ventral diencephalon is associated with freezing of gait in Parkinson’s disease: analysis of two cohorts
Source: NPJ Parkinsons Dis. 2025 Mar 6;11:44. doi: 10.1038/s41531-025-00893-5 (PMC11885666; doi:10.1038/s41531-025-00893-5)

**Supplementary Material**

**Supplementary Table 1**. Comparison between the included and excluded subjects

|  | Included (N = 94) | Excluded (N = 329) | *P* value |
| --- | --- | --- | --- |
| Age at admission, years, median (IQR) | 60.8 (53.5–67.9) | 62.85 (55.75–69.09) | 0.160 |
| Sex, n (F/M) | 28/66 | 118/211 | 0.274 |
| Years of education, years, median (IQR) | 16 (14–18) | 16 (14–18) | 0.861 |
| Duration of disease, years, median (IQR) | 4 (2.6–7.0) | 4.37 (2.48–8.43) | 0.992 |
| Hoehn-Yahr stage, median (IQR) | 2 (1–2) | 2 (1–2) | 0.463 |
| MDS-UPDRS I, median (IQR) | 4 (2–6) | 5 (3–8) | **0.002** |
| MDS-UPDRS II, median (IQR) | 4 (2–7) | 5 (3–8) | **0.023** |
| MDS-UPDRS III, median (IQR) | 18 (14–27) | 20 (15–26) | 0.564 |
| MDS-UPDRS total score, median (IQR) | 29 (19–27) | 32 (24–41) | **0.047** |
| MoCA score, median (IQR) | 28 (27–29) | 27 (26–29) | **0.007** |
| DAT striatal binding ratios |  |  |  |
| Mean caudate uptake, median (IQR) | 1.92 (1.54–2.25) | 1.97 (1.66–2.37) | 0.139 |
| Mean putamen uptake, median (IQR) | 0.75 (0.63–0.96) | 0.80 (0.63–0.97) | 0.585 |
| Cerebrospinal fluid biomarkers |  |  |  |
| α-syn, pg/mL, median (IQR) | 1374.10 (1007.10–1754.55) | 1409.90 (1059.60–1797.55) | 0.342 |
| Aβ42, pg/mL, median (IQR) | 781.50 (575.20–1022.00) | 864.40 (622.50–1134.00) | 0.131 |
| Phosphorylated Tau, pg/mL, median (IQR) | 12.74 (10.53–16.99) | 13.74 (11.38–17.57) | **0.035** |
| Total Tau, pg/mL, median (IQR) | 149.00 (124.35–192.50) | 159.65 (129.80–206.33) | 0.110 |

Abbreviations: DAT, dopamine transporter; MDS-UPDRS, movement disorder society united Parkinson’s disease rating scale; MoCA, Montreal Cognitive Assessment.

**Supplementary Table 2**. Linear regression analysis for the association between VDC volume and FOG severity in cohort 1

|  | Model 1 | | Model 2 | |
| --- | --- | --- | --- | --- |
|  | Unstandardized β (95% CI) | P value | Unstandardized β (95% CI) | P value |
| Age | 0.018 (-0.001–0.038) | 0.063 | 0.004 (-0.013–0.021) | 0.616 |
| Sex | 0.329 (-0.066–0.724) | 0.102 |  |  |
| BMI | 0.041 (-0.021–0.102) | 0.195 |  |  |
| Disease duration | 0.007 (0.004–0.010) | **<0.001** | 0.002 (-0.001–0.005) | 0.268 |
| Hoehn-Yahr stage | 0.914 (0.698–1.131) | **<0.001** | 0.539 (0.280–0.798) | **<0.001** |
| Levodopa daily dosage at admission | 0.002 (0.001–0.002) | **<0.001** | 0.001 (0.000–0.001) | **0.040** |
| MDS-UPDRS III | 0.033 (0.024–0.043) | **<0.001** | 0.017 (0.007–0.028) | **0.001** |
| MMSE | -0.026 (-0.078–0.026) | 0.325 |  |  |
| MoCA | -0.032 (-0.071–0.008) | 0.113 |  |  |
| HAMA | 0.014 (-0.019–0.046) | 0.413 |  |  |
| HAMD | 0.010 (-0.017–0.037) | 0.469 |  |  |
| Mean VDC | 0 (-0.001–0) | 0.039 | 0 (-0.001–0) | 0.159 |

Model 1 was adjusted

Model 2 adjusted for variables showing a trend toward significant correlation with FOG severity (P<0.1) in the univariate linear regression model

Abbreviations: BMI, body mass index; CI, confidence interval; HAMA, Hamilton anxiety scale; HAMD, Hamilton depression scale; MDS-UPDRS, movement disorder society united Parkinson’s disease rating scale; MMSE, mini-mental state examination; MoCA, Montreal cognitive assessment; VDC, ventral diencephalon.

**Supplementary Table 3.** Linear regression analysis for the association between VDC volume and FOG severity in PPMI cohort

|  | Model 1 | | Model 2 | |
| --- | --- | --- | --- | --- |
|  | Unstandardized β (95% CI) | P value | Unstandardized (95% CI) | P value |
| Age | 0.033 (0.009–0.056) | **0.007** | 0.010 (-0.015–0.036) | 0.417 |
| Disease duration | 0.035 (0.001–0.069) | **0.046** | 0.012 (-0.021–0.046) | 0.461 |
| Hoehn-Yahr stage | 0.315 (-0.133–0.764) | 0.165 |  |  |
| MDS-UPDRS III | 0.032 (0.010–0.054) | **0.005** | 0.011 (-0.013–0.034) | 0.365 |
| MoCA | -0.047 (-0.152–0.059) | 0.380 |  |  |
| PIGD score | 0.110 (-0.079–0.299) | 0.250 |  |  |
| Mean caudate uptake | -0.420 (-0.844–0.004) | 0.052 | -0.383 (-0.810–0.044) | 0.078 |
| CSF Aβ42 | 0 (-0.001–0.000) | 0.330 |  |  |
| VDC volume at year four | -0.001 (-0.001–0) | **0.005** | -0.001 (-0.001–0) | **0.032** |

Model 1 was adjusted

Model 2 adjusted for variables showing a trend toward significant correlation with FOG severity (P<0.1) in the univariate linear regression model

Abbreviations: CI, confidence interval; CSF, cerebrospinal fluid; PIGD, postural instability gait difficulty; MDS-UPDRS, movement disorder society united Parkinson’s disease rating scale; MoCA, Montreal cognitive assessment; VDC, ventral diencephalon.

**Supplementary Fig. 1** Flowchart of patient selection in PPMI cohort


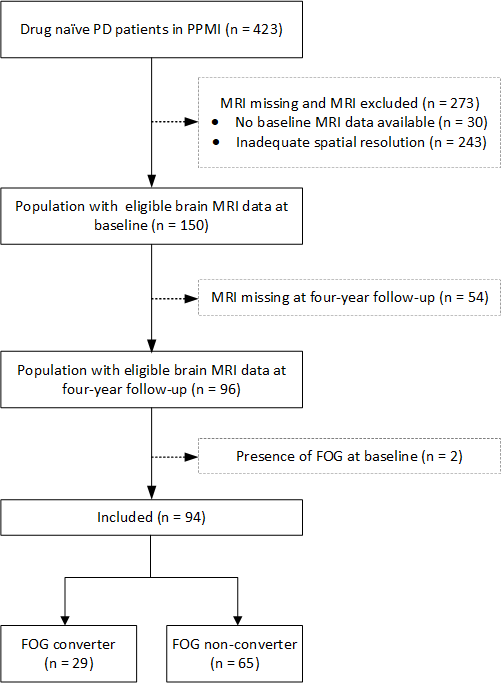


**Supplementary Fig. 2** Matrix of Principal Components

Seventeen principal components (PCs) were generated out of 89 parameters. PC1 accounted for 30.94% variance and was mainly composed of cortical thickness; PC2 accounted for 10.71% variance and was mainly composed of subcortical volumes.


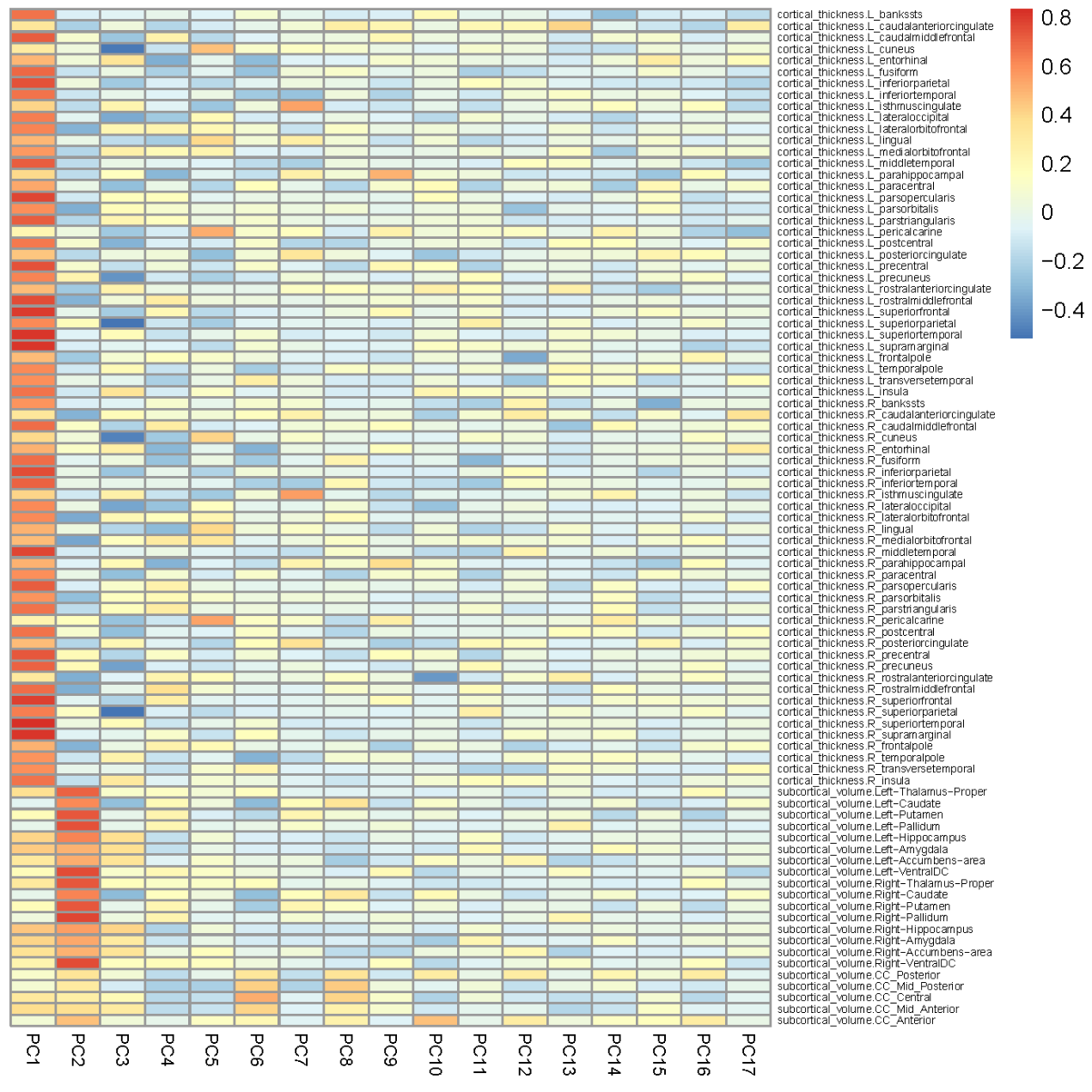

Supplement: Supplementary file 1 — Supplementary Material [file 41531_2025_893_MOESM1_ESM.docx]
